# Excoecarianin, Isolated from *Phyllanthus urinaria* Linnea, Inhibits Herpes Simplex Virus Type 2 Infection through Inactivation of Viral Particles

**DOI:** 10.1093/ecam/nep157

**Published:** 2011-05-26

**Authors:** Hua-Yew Cheng, Chien-Min Yang, Ta-Chen Lin, Liang-Tzung Lin, Lien-Chai Chiang, Chun-Ching Lin

**Affiliations:** ^1^Department of Cosmetic Applications & Management, Tung Fang Institute of Technology, 829 Kaohsiung County, Taiwan; ^2^School of Pharmacy, College of Pharmacy, Kaohsiung Medical University, 807 Kaohsiung, Taiwan; ^3^Department of Cosmetology and Health Care, Min Hwei College of Health Care Management, 736 Tainan County, Taiwan; ^4^Department of Nursing, Central Taiwan University of Sciences and Technology, 406 Taichung, Taiwan; ^5^Department of Microbiology & Immunology, Dalhousie University, Halifax, Nova Scotia, Canada B3H 1X5; ^6^Department of Microbiology, College of Medicine, Kaohsiung Medical University, 807 Kaohsiung, Taiwan

## Abstract

*Phyllanthus urinaria* Linnea (Euphorbiaceae) is one of the traditional medicinal plants widely used by oriental people to treat various diseases. We have previously demonstrated that the acetone extract of *P. urinaria* inhibits herpes simplex virus type 2 (HSV-2) but not HSV-1 infection. In a continuing effort to clarify the antiviral mechanisms of *P. urinaria*, we isolated the pure compound excoecarianin from the whole plant of *P. urinaria* through acetone extraction, and investigated its anti-HSV-1 and HSV-2 activities. Our results indicated that excoecarianin protected Vero cells from HSV-2 but not HSV-1 infection, and its 50% inhibitory concentration (IC_50_) was 1.4 ± 0.1 **μ**M. The antiviral effective concentration of excoecarianin did not affect the viability or the morphology of Vero cells. Although excoecarianin inhibited HSV-2 infection, the inhibitory effect, however, was most prominent when excoecarianin was concurrently added with the virus. Pretreatment of Vero cells with excoecarianin with removal of the drug prior to infection did not yield any antiviral effects, and the same observation was made for post viral entry treatment. Subsequent studies revealed that excoecarianin inactivated HSV-2 virus particles to prevent viral infection. A synergistic antiviral effect against HSV-2 was also observed when Vero cells were treated with a combination of acyclovir (ACV) and excoecarianin. These results suggested that excoecarianin merits to be further explored as an entry inhibitor against HSV-2 and could potentially be investigated for combinatorial drug treatment with nucleoside analogues such as ACV in therapeutic management of HSV-2 infection.

## 1. Introduction

Herpes simplex virus type 1 (HSV-1) and type 2 (HSV-2) are two species of the *Herpesviridae* family, which consists of large DNA-enveloped viruses. While HSV-1 is more frequently associated with oral–facial mucocutaneous lesions (cold sores and fever blisters), HSV-2 is more commonly associated with genital herpes. The two viruses can cause a variety of diseases, which in certain cases, may lead to complications, especially in immunocompromised patients. Herpes viruses usually produce lifelong infections, and recurrent infection may occur upon viral reactivation through stimuli such as sunlight, stress and weakened immunity.

Nucleoside analogs such as acyclovir (ACV) are the primary candidates used to treat HSV infections. Although some non-nucleoside inhibitors of herpesviruses have been developed [1–3], few of them are officially approved for HSV therapy [4, 5]. The lack of approved therapeutics and the emergence of ACV-resistant HSV strains have made the management of HSV infection a challenge, particularly in immunocompromised patients. In fact, previous reports have indicated that ACV-resistant HSV is more frequently identified in immunocompromised individuals rather than in those who are immunocompetent [6, 7]. Consequently, there is still a need to search for novel and more effective antiviral agents to prevent and/or to treat HSV infections [8–10].

Many naturally occurring plants, either as extracts or as pure compounds, have been reported to exhibit antiviral activities [11–14], indicating that plant materials may serve as a source for the discovery of antiviral agents. *Phyllanthus urinaria* Linnea (Euphorbiaceae) is a traditional medicinal plant that is widely used in Eastern Asia to treat a variety of diseases. The pharmacological activities of *P. urinaria* have been well documented in literature [15–36], and include antiviral activities [37–43] as well.

In our previous studies, we showed that the acetone, ethanol and methanol extracts of *P. urinaria* inhibited HSV infections *in vitro* [44]. Further isolation of gemin D, geraniin, hippomanin A and 1, 3, 4, 6-tetra-*O*-galloyl-*β*-d-glucose from the acetone extract of *P. urinaria* was found to suppress HSV infections at different magnitudes of potency [45, 46]. In a continuing effort to clarify the antiviral profile of *P. urinaria*, we isolated another pure compound, excoecarianin, from the whole plant of *P. urinaria* by acetone extraction, and investigated its anti-HSV-1 and HSV-2 activities.

## 2. Methods

### 2.1. Plant Materials

The plant *P. urinaria* was collected from Ping-Tung County and was authenticated using morphological and anatomical techniques. A voucher specimen, with reference number of KMU-HL-PUL 2002, was deposited at the Herbarium of the Graduate Institute of Natural Products of Kaohsiung Medical University.

### 2.2. Isolation of Excoecarianin from *P. urinaria*

**Figure 1:**
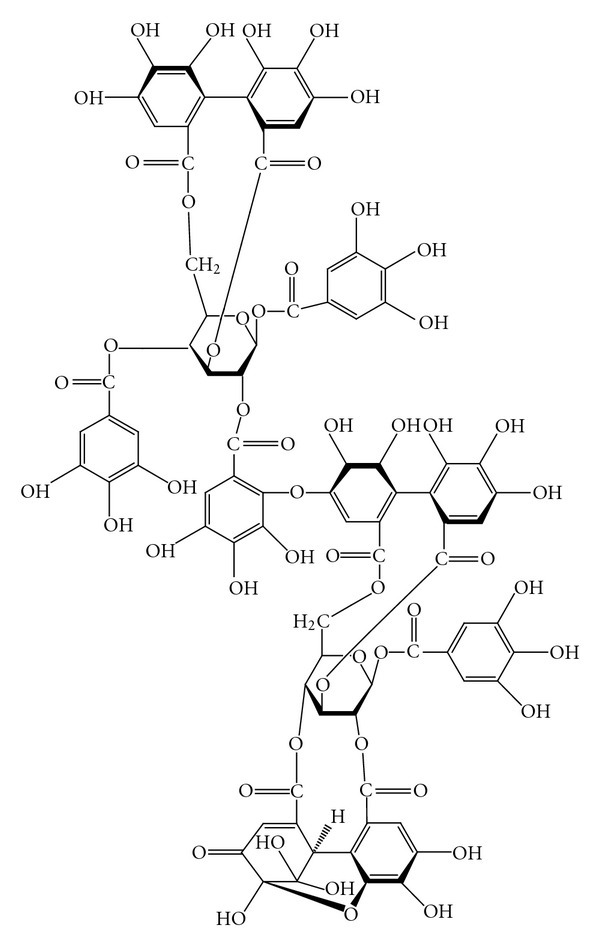
Chemical structure of excoecarianin.

Total 9.5 kg of fresh whole *P. urinaria* plant was cut into small pieces and extracted with acetone–water (4 : 1, v/v). The extract was concentrated under reduced pressure and then filtered. The filtrate was subsequently eluted with water–methanol and then with water–acetone through Sephadex LH-20 to give four fractions. Fraction 4 was further chromatographed on Sephadex LH-20, MCI-gel CHP 209, Fuji gel ODSG3, Sephadex LH-20 and Bondapack C18/porasil B to get 101 mg of excoecarianin (Figure 1) with the yield of 0.001%. The structure and purity (>95%) of excoecarianin were determined by spectroscopic and physical data analysis [47].

ACV and excoecarianin were dissolved in dimethyl sulfoxide (DMSO) and then diluted with sterile de-ionized distilled water before use. The final concentration of DMSO was <0.1%, which was not toxic to Vero cells as shown previously [44]. A 0.1% DMSO solution was included as control during the experiments.

### 2.3. Cell and Viruses

African green monkey kidney cells (Vero) (ATCC CCR-81) were used for antiviral assays. Vero cells were propagated in DMEM (Gibco, Invitrogen, USA) containing 5% of fetal calf serum (FCS). HSV-1 KOS strain and HSV-2 196 strain were grown in Vero cells. The viral titer was determined by plaque assay according to previously described procedures [48] and was expressed as plaque forming units (pfu) per milliliter. Virus stocks were stored at –80°C until use.

### 2.4. Cytotoxic Assay

Excoecarianin was tested for its cytotoxic effect on Vero cells by XTT (Sodium 3′-[1-(phenylamino-carbonyl)-3, 4-tetrazolium]-bis (4-methoxy-6-nitro) benzene sulfonic acid) (Sigma-Aldrich, USA) assay as described previously [45]. Briefly, Vero cells were seeded onto 96-well culture plates (Falcon, BD Biosciences, USA) at 1 × 10^4^ cells per well. After 4 h of incubation to allow the cells to settle, excoecarianin was added into each well at various concentrations. The plate was then incubated at 37°C in an atmosphere of 5% CO_2_ for 72 h. Later, the medium was discarded, and the cells were subsequently washed with phosphate buffered saline (PBS). The XTT reagent at a concentration of 300 *μ*g/mL was added, and the plate was re-incubated at 37°C for an additional 2 h to allow the development of formazan. The optical densities (ODs) were then measured with enzyme immunoassay (EIA) reader (Lab Systems MTX Labs, USA) at a test wavelength of 492 nm and a reference wavelength of 690 nm. The cytotoxic effect of excoecarianin on Vero cells was evaluated according to the OD readings obtained and the 50% cytotoxic concentration (CC_50_) was calculated as previously described [45, 46]. Besides cytotoxic cell death, Vero cells were also monitored in the presence of excoecarianin for changes in cellular morphology for up to 7 days.

### 2.5. Antiviral Assay

The inhibitory effect of excoecarianin on HSV infection was investigated by plaque reduction assay [49]. Vero cells were seeded onto 24-well culture plates (Falcon, BD Biosciences, USA) at a density of 1 × 10^5^ cells per well and incubated for 48 h to reach at least 95% confluency. The medium was then aspirated, and the cell monolayer was infected with 100 pfu of HSV-1 or HSV-2 in the absence or presence of excoecarianin. After 1 h incubation to allow virus adsorption, the cell monolayer was overlaid with overlay medium containing 1% of methylcellulose. The plate was further incubated at 37°C in an atmosphere of 5% CO_2_ for 48 h. Later, the overlay medium was removed, and the infected cell monolayer was fixed with 10% formalin. The cell monolayer was then stained with 1% crystal violet. The fraction of percent inhibition in inhibiting HSV infection was determined, and the minimal concentration that inhibited the formation of virus plaque number by 50% (IC_50_) was calculated [49].

### 2.6. Time-of-Addition Study

The time-of-addition effect of excoecarianin was examined according to the previously described procedures with some modifications [49]. Briefly, Vero cells were seeded onto 24-well culture plates (Falcon, BD Biosciences, USA) at a density of 1 × 10^5^ cells per well and incubated for 48 h to reach at least 95% confluency. Excoecarianin, at concentration of 1.5 *μ*M, was then added onto the cells at either before (–6 and –2 h), during (0 h) or after (2, and 4 h) HSV-2 infection times. For pre-infection (–6 and –2 h), cells were washed thrice by PBS to eliminate excoecarianin prior to the inoculation of the virus. Similar procedures as described in “antiviral assay” of Section 2 were carried to assess the virus plaques formed.

### 2.7. Viral Inactivation Assay

The direct effect of excoecarianin on HSV-2 infectivity was evaluated according to previously described procedures [50] with some modifications. Briefly, different concentrations (0.1, 0.5, 1.0, 1.5, 2.0, 2.5, and 4.0 *μ*M) of excoecarianin were mixed thoroughly with 1 × 10^5^ pfu of HSV-2. The mixture was then incubated at 37°C for 6 h. After the incubation, the mixture was diluted at least 100-fold and the residual virus infectivity was determined by plaque assay as described earlier.

### 2.8. Combined Treatment of ACV and Excoecarianin in Inhibiting HSV-2 Infection

The antiviral activity of excoecarianin in combination with ACV against HSV-2 was evaluated as described previously with some modifications [51–54]. The XTT assay was conducted as described above except that HSV-2 at multiplicity of infection (moi) of 1.0 was added concurrently with excoecarianin to the Vero cell monolayer. Inhibitory effect from the combination of ACV and excoecarianin against HSV-2 infection was analyzed by using the isobologram method. The IC_50_ was used to calculate the fractional inhibitory concentration (FIC) according to the formula that previously shown [51–54]. The interaction between excoecarianin and ACV was interpreted according to the combined FIC index (FIC of excoecarianin plus FIC of ACV). When the combined FIC index is equal to 1, the combination is assumed to act in an additive manner; when it is <1 the interaction is synergistic; and when the combined FIC index is >1 the interaction is antagonistic.

### 2.9. Statistical Analysis

Data are presented as mean ± SD of three independent experiments. The IC_50_ and CC_50_ values were calculated by Microsoft Excel 2003. The significance between the test sample and solvent control was analyzed by one-way analysis of variance (ANOVA) followed by Scheffe multiple comparison test. A *P* value < .05 was considered to be statistically significant.

## 3. Results

### 3.1. Cytotoxicity of Excoecarianin on Vero Cells

**Figure 2:**
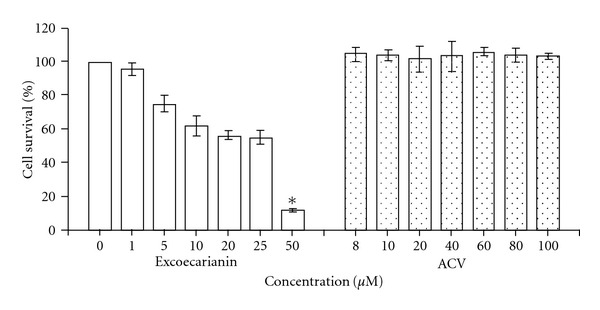
The cytotoxic effect of excoecarianin (open bars) and ACV (dotted bars) toward Vero cells as determined by XTT assay. Various concentrations of excoecarianin or ACV were added to Vero cells. After 72 h of incubation, the XTT solution was added and then the optical densities were measured. The cytotoxic effect of excoecarianin and ACV were evaluated and the 50% cytotoxic concentration (CC_50_) was calculated. Each bar represents the mean ± SD of three independent experiments. The asterisk indicates significant difference between test sample and solvent control (*P* < .05).

**Figure 3:**
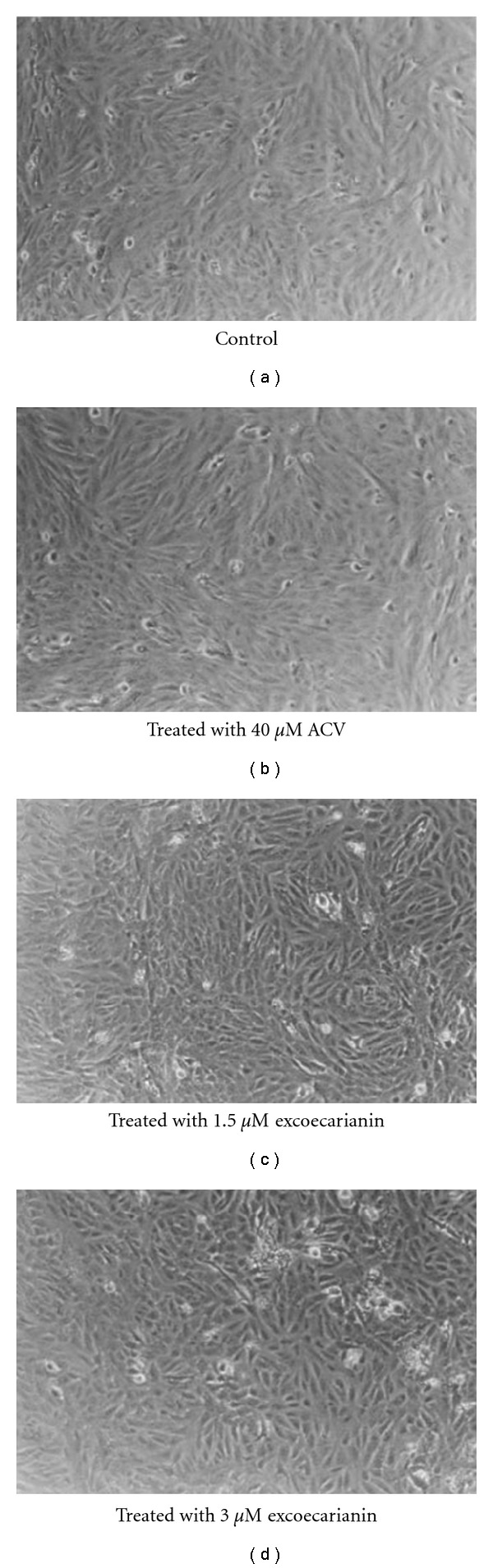
The morphology of Vero cells without treatment (a) and with treatment of ACV (b) or excoecarianin (c)-(d). Vero cells were seeded onto 24-well culture plates at density of 1 × 10^3^ cells per well. After 4 h, excoecarianin or ACV was added. The cells were incubated for 7 days, and the cellular morphology was examined under phase-contrast microscope.

The cytotoxic effect of excoecarianin toward Vero cells was investigated by the XTT method. Excoecarianin exhibited cytotoxic effect toward Vero cells in a concentration-dependent manner (Figure 2) with only 11.57% of cells surviving after treatment at 50 *μ*M (*P* < .05), but at least 75.2% and 95.4% cells remained alive at concentrations of 5.0 and 1.0 *μ*M, respectively. The CC_50_ of excoecarianin was 28.0 ± 2.6 *μ*M. No significant changes in cellular morphology were observed in excoecarianin-treated cells at a concentration of 3 *μ*M and lower (Figure 3). Overall, the results indicated that excoecarianin showed little cytotoxic effect at concentrations <5.0 *μ*M, and therefore, subsequent studies were performed with excoecarianin treatment at concentrations <5.0 *μ*M.

### 3.2. Antiviral Activity of Excoecarianin against HSV-1 and HSV-2 Infection

**Figure 4:**
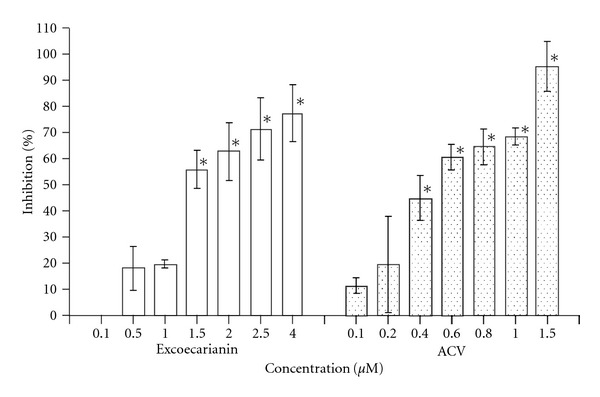
Inhibitory effect of excoecarianin (open bars) and ACV (dotted bars) against HSV-2 infection in Vero cell as determined by plaque reduction assay. Vero cells were incubated with 100 pfu of HSV-2 and different concentrations of excoecarianin or ACV. After 1 h, an overlay medium containing 1% methylcellulose was added. On Day 3 post-infection, the cell monolayer was stained with crystal violet and the virus plaques formed were counted. The percentage of inhibition was calculated by comparing the plaque number of compound-treated group to that of the untreated group. The concentrations of excoecarianin and ACV that inhibited 50% of HSV-2 infection (IC_50_) were determined. Each bar represents the mean ± SD of three independent experiments. The asterisk indicates significant difference between test sample and solvent control (*P* < .05).

The anti-HSV-1 and HSV-2 activities of excoecarianin were evaluated by plaque reduction assay. Excoecarianin inhibited HSV-2 infection in a concentration-dependent manner (FIgure 4). The percentage of inhibition were 0.02 ± 0.02%, 18.3 ± 8.5%, 20.0 ± 1.5%, 56.2 ± 7.3%, 62.8 ± 11.0%, 71.4 ± 11.9% and 77.5 ± 10.8% at the concentrations of 0.1, 0.5, 1.0, 1.5, 2.0, 2.5, and 4.0 *μ*M, respectively. The IC_50_ of excoecarianin against HSV-2 infection was determined to be 1.4 ± 0.1 *μ*M. When tested against HSV-1 infection, excoecarianin was not able to suppress HSV-1 infection (data not shown). At the highest concentration used (4.0 *μ*M), excoecarianin could only inhibit 11% of HSV-1 infection.

The selectivity index (SI) measures the safety of a compound to be used as antiviral agent and also confirms that the antiviral effect of a compound is not related to its toxic effect on the cell, is calculated by dividing the CC_50_ with the IC_50_ value of the test compound. The SI of excoecarianin was 20.0 against HSV-2 infection.

### 3.3. Antiviral Activity of Excoecarianin at Different Times of Addition

To study the inhibitory effect of excoecarianin on the stage of HSV-2 infection, the compound was added at different periods (before, during and after) of virus infection. Results showed that excoecarianin added at 2 or 6 h prior to virus infection, and then being removed by PBS washes before the infection, did not have any antiviral activity. When added concurrently with HSV-2, excoecarianin, at concentration of 1.5 *μ*M, inhibited 56.2% of virus infection. The inhibitory rate, however, declined to 22.6 and 31.2% when added at 2 and 4 h after infection, respectively (Table 1). This observation indicated that excoecarianin effectively inhibited HSV-2 infection only when it is present at the time of the infection, suggesting its antiviral activity is likely to be at the viral entry stage.

### 3.4. Mechanism of Action of Excoecarianin against HSV-2 Infection

To investigate whether excoecarianin inhibited HSV-2 through inactivation of viral particles, a suspension of the virus was treated at 37°C for 6 h with various concentrations of excoecarianin. The residual viral infectivity was determined by plaque assay. As shown in Table 2, excoecarianin inactivated viral infectivity in concentration-dependent manner. While it did not inactivate HSV-2 at concentration of ≤1.0 *μ*M, the residual viral infectivity gradually declined from 61.3 ± 7.8%, to 27.7 ± 7.9%, and to 36.3 ± 10.8% as the concentrations of excoecarianin increased from 1.5, to 2.0, and to 2.5 *μ*M, respectively. Excoecarianin drastically reduced viral infectivity at concentration of 4.0 *μ*M with only 1.5 ± 0.1% of virus being able to produce infectivity (*P* < .05). In contrast, ACV, a well-defined anti-HSV-2 agent which specifically inhibits viral DNA synthesis, did not have effect on virus infectivity even at a concentration of 10.0 *μ*M.

### 3.5. Combined Treatment of ACV and Excoecarianin against HSV-2 Infection

**Figure 5:**
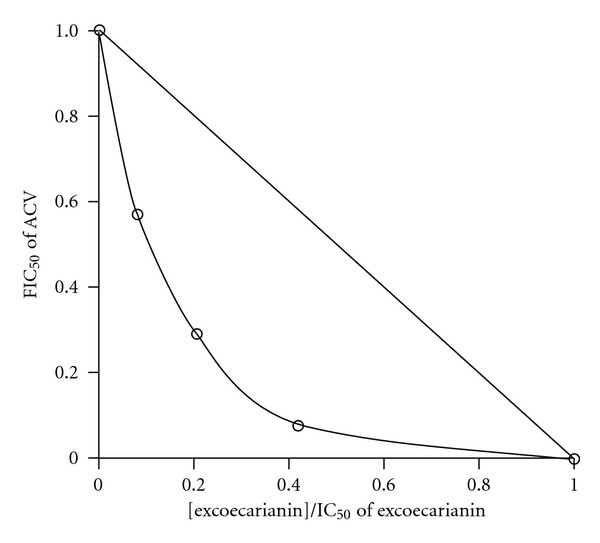
Synergistic antiviral activity of ACV and excoecarianin in Vero cells. IC_50_ values were derived from the data shown in Table 3 and used to construct the isobologram. FIC_50_ of ACV represents the ratio of the IC_50_ of ACV in the presence of a constant concentration of excoecarianin to the IC_50_ of ACV alone. The *x*-axis represents the ratio of the fixed concentration of excoecarianin to the IC_50_ of excoecarianin alone. In this representation, displacement of the experimental data points to the left of the theoretical line is indicative of synergistic behavior.

The inhibitory effect of ACV plus excoecarianin against HSV-2 infection in Vero cells was examined by XTT assay. The inhibitory activity was further evaluated by the isobologram method. Low concentrations of ACV could inhibit HSV-2 infection by the addition of excoecarianin (Table 3). The IC_50_ value for ACV could be reduced from 1.33 ± 0.84 *μ*M of ACV alone to 0.76 ± 0.34, 0.39 ± 0.18 and 0.11 ± 0.02 *μ*M of ACV plus 1.0, 2.5 and 4.0 *μ*M of excoecarianin, respectively. The FICs of ACV plus excoecarianin were in the range 0.50–0.66, indicating that the combinatorial effect of ACV plus excoecarianin in inhibiting HSV-2 infection was synergistic (Table 3 and Figure 5). None of these drug combinations exhibited cytotoxic effect against Vero cells at the concentrations used as assessed by cytotoxicity assay (data not shown).

## 4. Discussion

Our present study demonstrated that excoecarianin inhibited HSV-2 infection but not HSV-1 infection. This antiviral effect was not correlated to cellular cytotoxicity as the effective micromolar concentrations of excoecarianin used did not affect cell viability (Figure 2) or induce changes in cellular morphology (Figure 3). The antiviral activity was most prominent when excoecarianin was added during viral infection. This effect was attributed to the compound's ability to directly reduce viral infectivity through interaction with HSV-2 viral particles. In addition, combined treatment of ACV and excoecarianin synergistically inhibited HSV-2 infection.

Our time-point assessment of antiviral activity indicated that excoecarianin most effectively inhibited HSV-2 infection only when it was concurrently present with the virus at the time of infection, but was less effective when cells were pre-treated with it (and washed) or treated post viral entry. Since our pre-treatment protocol involved washing the compound off prior to infection, these results would speculate that excoecarianin probably does not prevent HSV-2 infection by masking cell surface HSV-2 receptors through covalent bonds formation to remain attached, nor does it trigger any cellular antiviral responses including induction of type I interferons to control the viral infection. Clearly, excoecarianin is affecting the early stage of HSV-2 infection and the presence of the drug at the time of the infection is crucial. The observation suggests that the inhibition on HSV-2 by excoecarianin was most likely mediated by interfering with virus entry step without having any direct effect on the cell itself, and indeed it was confirmed by our subsequent data that excoecarianin directly inactivated HSV-2 particles (Table 2). This effect is different from that of ACV, which acts as an inhibitor of HSV DNA synthesis. The ability of excoecarianin to inactivate HSV-2 virus particles thus makes it useful as an anti-HSV-2 agent in preventing *de novo* viral infection and thereby could help control viral spread and limit recurrent infections.

In virus taxonomy, HSV-1 and HSV-2 are members of the genera *Simplexvirus* from the subfamily *Alphaherpesvirinae* in *Herpesviridae* family [55]. Both viruses are sensitive to nucleoside-like drugs, such as ACV. The effectiveness on inhibiting HSV-2 but not HSV-1 infection suggested that excoecarianin is specific in mediating its anti-HSV activity. As HSV-1 and HSV-2 virions share high degree of similarity, it is unclear at the moment how excoecarianin is specific only to HSV-2. Excoecarianin could possibly be specific to viral glycoproteins expressed by HSV-2 such as glycoprotein C (gC-2) which has been shown to contribute to serotype differences in cell tropism and may play a different role with respect to its HSV-1 counterpart gC-1 [56, 57]. Further studies are required to clarify the underlying reason(s) of the selectivity in anti-HSV effects mediated by excoecarianin.

Our previous studies also revealed that the acetone extract and several pure compounds found in the extracts from *P. urinaria* could inhibit HSV-2 infection at different magnitudes of potency [44–46]. Particularly, we have observed that the acetone extract of *P. urinaria* inhibited HSV-2 infection through diminishing the virus infectivity, and the acetone extract was only active in inhibiting HSV-2 when added concurrently with the virus [44]. The acetone extract also did not have any direct effect on the cells in preventing viral infection as indicated by a lack of benefit from pre-treatment dosage [44]. Based on our current experimental observations, the acetone-isolated excoecarianin from *P. urinaria* is likely responsible, at least in part, for the previous antiviral effects observed from the *P. urinaria* acetone extract. Furthermore, the identification of excoecarianin as a specific HSV-2 entry inhibitor by our study suggests that excoecarianin or the standardized acetone extract *P. urinaria* could both be explored for therapeutic development against HSV-2 infection. As *P. urinaria* also possesses antiviral activity against other viruses as well, including hepatitis B virus [37–41], retrovirus [42] and Epstein-Barr virus [43], it is unclear whether excoecarianin can mediate similar antiviral activities against other viruses. It would also be interesting to investigate whether excoecarianin or the standardized extract could improve the disease pathogenesis in viral co-infection cases such as in immunocompromised patients as an adjunct therapy.

We have demonstrated here that the combined treatment of ACV and excoecarianin exhibits synergistic activity in inhibiting HSV-2 infection, decreasing the concentration required for ACV to attain similar antiviral profile. This result has clinical significance in the management of HSV-2 infection. As there are more ACV-resistant HSV related cases being identified from immunocompromised patients as a result of the emergence of HIV or organ transplantation [6, 7, 58], anti-HSV agents targeting viral enzymes or factors essential for entry will likely be useful for controlling nucleoside-resistant strains [59]. The application of excoecarianin would complement ACV treatment, not only in lowering the amount of drug necessary to achieve viral eradication but would also help decrease the risk of ACV-resistance from occurring. Thus, the difference in mechanism of action between excoecarianin and ACV, along with the synergistic effect observed from their combined treatment, implies that excoecarianin could be useful in therapy against HSV-2 infection in immunocompromised patients.

In summary, we have identified the acetone-extracted excoecarianin from *P. urinaria* as an antiviral compound that is specific for HSV-2, which may be responsible in part for the previously observed *P. urinaria*'s antiviral effect against this viral infection. The ability of excoecarianin to inactivate HSV-2 virus particles and to act synergistically with the nucleoside analogue ACV may make it useful to control viral spread in *de novo* and recurrent infections. We therefore suggest that the development of excoecarianin or the standardized extract of *P. urinaria* as an oral agent or as a topical cream may be further investigated as an effective strategy in managing HSV-2 infections.

## Funding

National Science Council grant [NSC 92-2320-B-037-044].

## Figures and Tables

**Figure 6:**
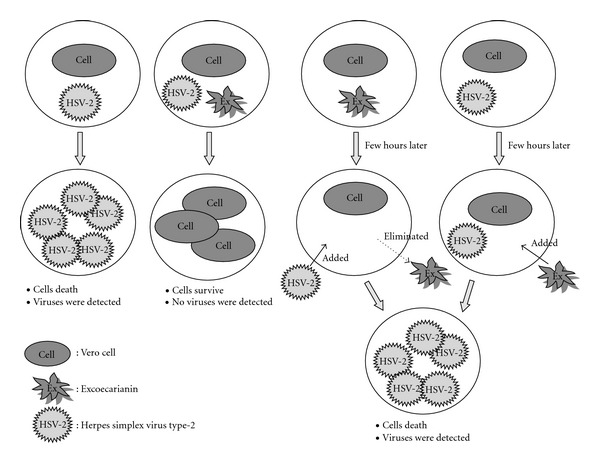
The time-of-addition effect of excoecarianin against HSV-2 infection. Excoecarianin added prior to virus infection and then washed out did not protect the cells from HSV-2 infection. The addition of excoecarianin after 2 h of the virus inoculation also did not inhibit HSV-2 infection. The compound can inhibit HSV-2 infection only when it was added concurrently with the virus infection.

**Table 1:** The effect of treatment time on the antiviral activity of excoecarianin.

Time periods of compound addition	Percentage of inhibition	
	Excoecarianin	ACV
Before infection		
–6	0.0 ± 0.0	0.0 ± 0.0
–2	0.0 ± 0.0	15.0 ± 7.6
During infection		
0	56.2 ± 7.3*	95.4 ± 1.7*
After infection		
2	22.6 ± 10.2	92.6 ± 5.0*
4	31.2 ± 19.2	94.9 ± 4.7*

Excoecarianin and ACV were tested at a concentration of 1.5 and 5 *μ*M, respectively; Data were mean ± SD of three independent experiments; *P < .05 (compared between control and tested compound).

**Table 2:** The effect of excoecarianin against HSV-2 infectivity.

	Concentration (*μ*M)	Percentage control of viral infectivity
Control	0	100.0 ± 0.0
Excoecarianin	0.1	91.6 ± 7.4
	0.5	98.8 ± 2.1
	1.0	96.2 ± 6.6
	1.5	61.3 ± 7.8*
	2.0	27.7 ± 7.9*
	2.5	36.3 ± 10.8*
	4.0	1.5 ± 0.1*

ACV	0.5	100.0 ± 0.5
	2.5	92.8 ± 6.5
	5.0	92.9 ± 4.9
	10.0	91.7 ± 8.2

Excoecarianin was mixed with HSV-2 at 37°C for 6 h before the residual viral titer was determined by plaque assay; Values were mean ± SD of three independent experiments; *P < .05 (compared between control and tested compound).

**Table 3:** Inhibitory effects of excoecarianin in combination with ACV against the infection of HSV-2 in Vero cells.

Compound(s)	Mean (IC_50_ ± SD)^a^	FIC_excoecarianin_ + FIC_ACV_^b^	FIC index interpretation
Excoecarianin alone	11.87 ± 2.9	—	—
ACV alone	1.33 ± 0.84	—	—
ACV + 1.0 *μ*M excoecarianin	0.76 ± 0.34	0.66	synergistic
ACV + 2.5 *μ*M excoecarianin	0.39 ± 0.18	0.51	synergistic
ACV + 5.0 *μ*M excoecarianin	0.11 ± 0.02	0.50	synergistic

^a^Results are based on three independent experiments; ^b^FIC_excoecarianin_ and FIC_ACV_ are the fractional inhibitory concentration (FIC) of excoecarianin and ACV, respectively.
